# Elevated CO_2_ increases energetic cost and ion movement in the marine fish intestine

**DOI:** 10.1038/srep34480

**Published:** 2016-09-29

**Authors:** Rachael M. Heuer, Martin Grosell

**Affiliations:** 1 University of Miami- Rosenstiel School of Marine and Atmospheric Science, 4600 Rickenbacker Causeway, Miami, FL 33149, USA

## Abstract

Energetic costs associated with ion and acid-base regulation in response to ocean acidification have been predicted to decrease the energy available to fish for basic life processes. However, the low cost of ion regulation (6–15% of standard metabolic rate) and inherent variation associated with whole-animal metabolic rate measurements have made it difficult to consistently demonstrate such a cost. Here we aimed to gain resolution in assessing the energetic demand associated with acid-base regulation by examining ion movement and O_2_ consumption rates of isolated intestinal tissue from Gulf toadfish acclimated to control or 1900 μatm CO_2_ (projected for year 2300). The active marine fish intestine absorbs ions from ingested seawater in exchange for HCO_3_^−^ to maintain water balance. We demonstrate that CO_2_ exposure causes a 13% increase of intestinal HCO_3_^−^ secretion that the animal does not appear to regulate. Isolated tissue from CO_2_-exposed toadfish also exhibited an 8% higher O_2_ consumption rate than tissue from controls. These findings show that compensation for CO_2_ leads to a seemingly maladaptive persistent base (HCO_3_^−^) loss that incurs an energetic expense at the tissue level. Sustained increases to baseline metabolic rate could lead to energetic reallocations away from other life processes at the whole-animal level.

Although fish respond effectively and restore blood pH during CO_2_ induced acid-base balance disturbance, recent studies have noted potential impacts of ocean acidification across a range of areas including neurosensory disruptions, increased otolith growth, altered mitochondrial function, and changes to metabolic rate^1^,^2^,^3^,^4^,^5^,^6^,^7^,^8^,^9^. A suggested unifying hypothesis is that the compensatory response induced during CO_2_ exposure to correct blood pH may have negative downstream consequences or induce tradeoffs^1^,^4^,^10^. This pH compensation is associated with a sustained increase of extra and intracellular concentration of HCO_3_^−^ in response to the elevated partial pressure of CO_2_ (*p*CO_2_)^11^,^12^,^13^,^14^,^15^. Assuming the rate of adaptation does not keep pace with a rapidly acidifying ocean, fish in future oceans will require consistent and elevated levels of ion exchange to sustain elevated HCO_3_^−^ and normal pH, a process that is anticipated to add to the cost of basic maintenance of homeostasis, most often quantified as standard metabolic rate (the minimum O_2_ consumption rate of a resting animal in the post-absorptive state^16^).

The gill, intestine, and kidney are the organs involved with acid-base balance and osmoregulation in marine fish^13^,^17^. Estimates vary widely, but metabolic cost of ion regulation has been proposed to range from 1–30% or 6–15% of whole-animal standard metabolic rate (reviewed in ref. 18,19). More targeted estimates of ion transport using specific isolated organs suggest that the gills and intestine account for ~4%^20^ and 5.6%^21^ of standard metabolic rate, respectively, which combined would account for about 10% of whole-animal standard metabolic rate. Interestingly, despite the need for HCO_3_^−^ retention during a CO_2_-induced acidosis, toadfish experience ~34% increase in HCO_3_^−^ ion loss through the intestine when exposed to near-future CO_2_ scenarios (1900 μatm CO_2_)^22^, a trend also apparent at higher CO_2_ levels^23^. Although the intestine is a metabolically active tissue central to water balance and survival in seawater, the functional consequence of this relatively high HCO_3_^−^ loss during CO_2_ acclimation has not been examined. Elevated HCO_3_^−^ secretion may also stimulate an increase in fish CaCO_3_ production and release to the marine environment, potentially altering the marine carbon cycle^24^. Since teleosts are the largest vertebrate group with a vital role in oceanic food webs, it is important to understand how impacts to the intestine during future ocean acidification scenarios could affect both fish and their surrounding environment. The first goal of the present study was to determine if the intestine of a marine teleost, an organ known to show phenotypic plasticity in other ion regulatory challenges^25^, would dynamically regulate intestinal function to reduce CO_2_-induced intestinal HCO_3_^−^ loss in favor of whole-body HCO_3_^−^ retention. We predicted that tissue from CO_2_-acclimated toadfish would show reduced bicarbonate secretion rates. After seeing a stimulation rather than a reduction of bicarbonate secretion rates, a second goal was to test the hypothesis that an increase in intestinal ion transport that occurs in response to elevated CO_2_ would be associated with an increased tissue metabolic demand. Experiments were conducted at 1900 μatm CO_2_, a level currently seen in certain coastal and upwelling zones^26^,^27^ and predicted globally in year 2300^28^.

## Results

### Bicarbonate secretion rates of isolated tissue

**Figure 1:**
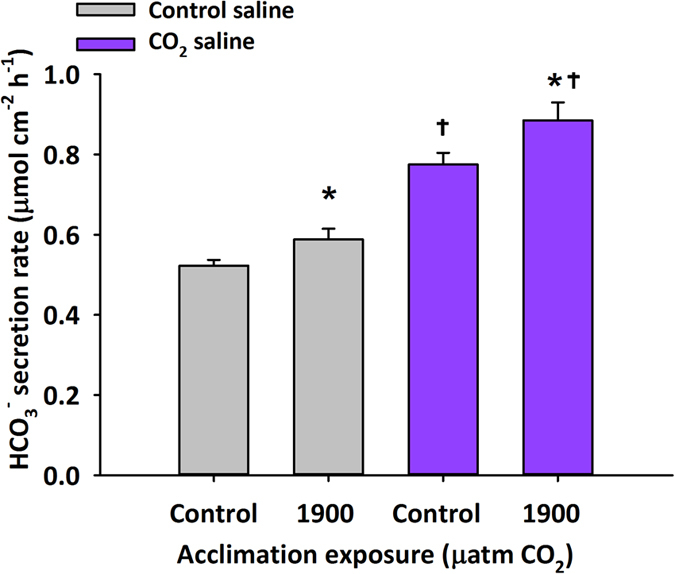
Bicarbonate secretion rates of isolated anterior intestine from control and CO_2_ acclimated toadfish. Effect of blood-side saline composition and acclimation exposure on HCO_3_^−^ secretion rates (means ± s.e.m.) of isolated anterior intestinal tissue obtained from gulf toadfish acclimated to control (~440 μatm CO_2_, n = 9) or ~1900 μatm CO_2_ (n = 10) for 2–4 weeks. Tissues from either control or 1900 μatm CO_2_ acclimated fish mounted in this dual chambered Ussing/pH-stat system were bathed on either side by salines that mimicked *in vivo* ionic composition. Each tissue received two blood-side (serosal) saline treatments, control and CO_2_ saline, representative of HCO_3_^−^ and *p*CO_2_ previously measured in toadfish blood following control and 1900 μatm CO_2_ exposure (Supplementary Table 3). Two-way ANOVA; Acclimation exposure: P < 0.004, Saline: P < 0.001, Acclimation exposure × saline: P < 0.475. *Significant acclimation effect, ^†^Significant saline effect.

Contrary to expectations, anterior intestinal tissue from CO_2_ acclimated toadfish exhibited significantly increased HCO_3_^−^ secretion rates (μmol cm^−2^ h^−1^) when compared to control tissue under identical conditions (Fig. 1). This result indicated prior acclimation to CO_2_ does not suppress but stimulates intestinal HCO_3_^−^ transport by around 13%. In addition, within both the control and CO_2_ acclimated fish, HCO_3_^−^ secretion rates using serosal salines mimicking plasma conditions at 1900 μatm CO_2_ were significantly higher than HCO_3_^−^ secretion rates under control serosal salines (Two-way ANOVA, Fish treatment P < 0.009, Saline P < 0.001, Fish treatment × saline P < 0.490, Fig. 1). TEP and conductance remained stable during experiments and were in agreement with earlier reports (Supplementary Table 1)^21^,^29^.

### Oxygen consumption rates of isolated tissue

**Figure 2:**
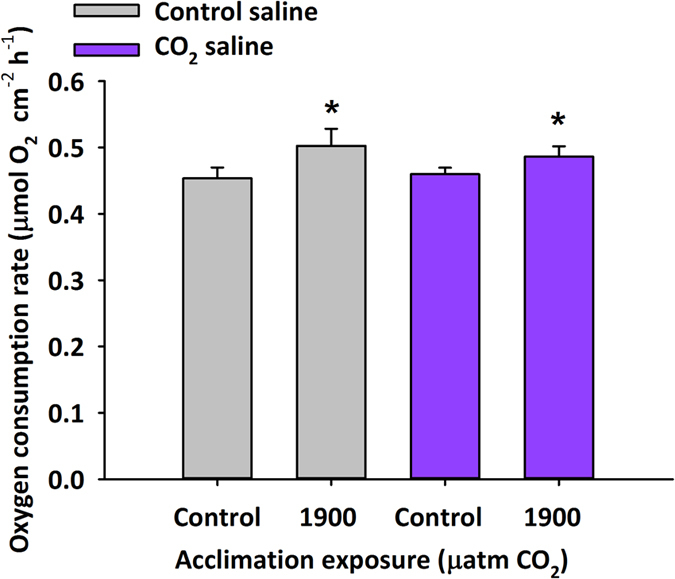
Oxygen consumption of isolated anterior tissue from control and CO_2_ acclimated toadfish. Effect of blood-side saline composition and acclimation exposure on oxygen consumption rates (means ± s.e.m.) of isolated anterior intestinal tissue taken from toadfish acclimated to control (~440 μatm CO_2_; n = 12) or ~1900 μatm CO_2_ (n = 8) for 2–4 weeks. Tissues from either control or 1900 μatm CO_2_ acclimated fish mounted in this dual-chambered epithelial respirometer were bathed on either side by salines designed to mimic *in vivo* ionic composition. Each tissue received two blood-side (serosal) saline treatments, control saline and CO_2_ saline, that were representative of HCO_3_^−^ and previously measured in toadfish blood following acclimation at control and 1900 μatm CO_2_ (Supplementary Table 3). Two-way ANOVA; Acclimation exposure: P < 0.033, Saline: P < 0.769, Acclimation exposure × saline: P < 0.509. *Significant acclimation effect.

Under both saline compositions, tissue from 1900 μatm CO_2_ acclimated toadfish showed a significant 8% increase in oxygen consumption rate compared to tissue from control acclimated fish. In contrast to HCO_3_^−^ secretion rates, oxygen consumption rates of isolated tissue from both control and 1900 μatm CO_2_ exposed fish showed no effect of saline composition (Two-way ANOVA, Fish treatment P < 0.033, Saline P < 0.769, Fish treatment × saline P value < 0.509, Fig. 2). A similar relationship was observed when data was corrected for body mass (Supplementary Fig. 1).

## Discussion

These results indicate that the marine fish intestine has a higher metabolic demand at 1900 μatm CO_2_ than at present-day ambient conditions (~400 μatm CO_2_), that is likely attributed to an increase in intestinal HCO_3_^−^ loss from the body. This elevated CO_2_ level is predicted for year 2300 and is currently seen in upwelling coastal areas^26^. Similar to other fish experiencing elevated CO_2_ in a marine environment, gulf toadfish have been shown to defend blood pH following exposure to 1900 μatm CO_2_ by sustaining elevated levels of HCO_3_^−^ in the face of higher *p*CO_2_ in the blood^11^. However, this compensation was associated with an increased intestinal HCO_3_^−^ loss that was presumed to be associated with an activation of existing ionoregulatory transport pathways^11^,^22^. These pathways involve the movement of plasma HCO_3_^−^ into the intestinal lumen in exchange for Cl^−^ and are critical for maintaining water balance^30^. However, from a whole-animal acid-base balance perspective, HCO_3_^−^ loss during CO_2_ compensation counteracts the need to retain HCO_3_^−^ and defend pH, suggesting that fish faced with longer term acclimation to CO_2_ must dynamically downregulate pathways involved in intestinal HCO_3_^−^ secretion. Contrary to our initial hypothesis, comparison of HCO_3_^−^ secretion rates of intestinal tissue from CO_2_ and control acclimated fish under identical saline conditions revealed that CO_2_ exposure leads to a stimulation, rather than a downregulation of HCO_3_^−^ transport pathways.

**Figure 3:**
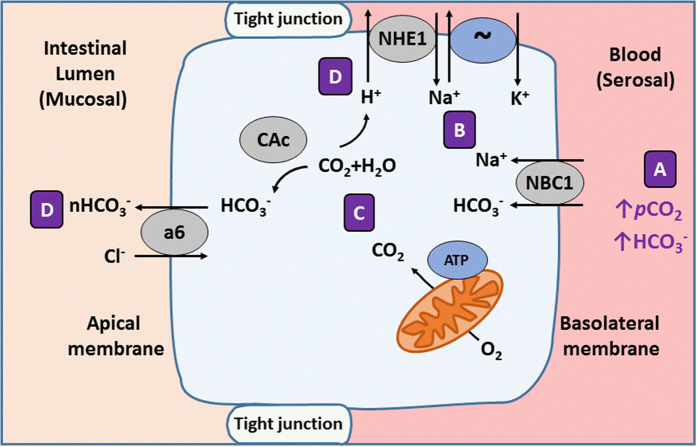
Proposed impacts of 1900 μatm CO_2_ on intestinal transport physiology in a marine teleost. Compensation for a CO_2_-induced acidosis increases HCO_3_^−^ and *p*CO_2_ in extracellular fluids^11^. (**A**) Elevated serosal HCO_3_^−^ during CO_2_ exposure stimulates transport via Na^+^:HCO_3_^−^ co-transporter, NBC1, leading to both an increase influx of HCO_3_^−^ and Na^+^ across the basolateral membrane. (**B**) Sustained influx of Na^+^ via NBC1 likely leads to a demand for increased Na^+^ extrusion via the energy-demanding Na^+^ K^+^ ATPase (~). (**C**) The metabolic demand and increased O_2_ consumption associated with handling an increased Na^+^ influx would generate additional endogenous CO_2_, increasing substrate for intracellular CAc hydration. (**D**) Intracellular HCO_3_^−^ generated via this process likely accounts for the observed stimulation of bicarbonate secretion via SLC26a6 (a6) in isolated tissue from fish acclimated to 1900 μatm CO_2_ compared to control fish under serosal salines with identical bicarbonate concentrations. Protons generated in via carbonic anhydrase in step C would likely be extruded from the cell via NHE1 and add to the increase in intracellular Na^+^. For a more detailed overview of marine fish intestinal transport processes see review^30^.

We propose that the 13% increase in HCO_3_^−^ loss from CO_2_ acclimated fish reflects an increased energetic demand, and thus increased CO_2_ production, in intestinal tissue. This suggestion is supported by an 8% increase in O_2_ consumption in tissue from CO_2_-acclimated fish. A detailed mechanistic explanation of this proposed response can be seen in Fig. 3. Sustained elevated plasma HCO_3_^−^ following CO_2_ compensation leads to an increase in HCO_3_^−^ movement from the blood into the intestinal cell that is paired with the movement of Na^+^ through basolateral NBC1, a Na^+^-HCO_3_^−^ co-transporter^31^. The sustained Na^+^ influx must be compensated for by Na^+^ extrusion through the Na^+^-K^+^ ATPase (NKA). Increased NKA activity leads to an increased ATP and thus O_2_ demand. Meeting this demand results in increased endogenous CO_2_ production available for hydration via intracellular carbonic anhydrase (CAc) to form additional HCO_3_^−^. Secretion of this excess HCO_3_^−^ through apical SLC26a6 anion exchange likely accounts for the stimulation of HCO_3_^−^ loss during CO_2_ exposure. Hydration of endogenous CO_2_ also results in formation of protons that must be eliminated from the intestinal cell and could put additional demand on the gill. This proton extrusion by the intestine may occur via Na^+^-dependent or independent pathways^30^, both of which are energy demanding and could contribute to the observed elevation of O_2_ consumption.

It is likely that the 8% increase in intestinal tissue O_2_ consumption would not be observable by measurements of whole-animal metabolic rate. Difficulties in picking up small differences in organismal metabolic rate may explain some of the varying results from studies examining the effects of ocean acidification on fish standard metabolic rate^1^. In addition, there appears to be considerable intra- and inter-species variation in the response to elevated CO_2_ and there is inherent difficulty in comparing measurements using different methodologies^32^,^33^. As demonstrated in the present study and by others^5^,^34^,^35^,^36^,^37^, increased resolution and mechanistic insight into energetic tradeoffs and/or apparent consequences of ocean acidification may be obtained by integrating techniques and methods that examine multiple levels of organization. Altered mitochondrial capacity^5^, shifts in energetic budgets^34^,^38^,^39^, increased expression/activity of gill and intestinal ionoregulatory genes and proteins^15^, increased ventilation^40^, and increased protein turnover^38^ have all been noted during ocean acidification-relevant CO_2_ exposures with little impact to whole-animal measurements. The importance of integrating multiple techniques is apparent in a recent study on CO_2_ exposure (1,200 and 2,200 μatm CO_2_) in Atlantic cod exhibiting increased intestinal NKA mRNA and protein concentration, while exhibiting no change in NKA protein activity^41^. While the present study of isolated intestinal tissue precluded normal hormonal cascades or feedback mechanisms, it offered the advantage of careful control of blood-side (serosal) saline conditions and made it possible to identify mechanistic differences in tissue function that were impossible to observe in previous *in vivo* work^22^. One other caveat to note is that air, rather than custom CO_2_/O_2_ gas mixtures were used during respirometry experiments. Earlier work on this preparation revealed that tissue metabolic rate is not limited by O_2_ above 75% air saturation^21^. Thus, tissue was not O_2_-limited in the present study since saturation remained above 80%.

The marine fish intestine is fine-tuned to changes in plasma HCO_3_^−^ to aid in water uptake and to handle the alkaline tide associated with digesting a meal^21^,^31^. Although the functional consequence of stimulated intestinal HCO_3_^−^ loss remains to be fully elucidated, the increase in O_2_ consumption leads us to conclude that this CO_2_-induced response is potentially maladaptive but persists for protection of osmoregulatory and digestive functions. Support for this conclusion comes from a recent study, on Atlantic cod, demonstrating that high levels of CO_2_ (9200 μatm) lengthen the time needed to digest a meal^42^. Interestingly, reduced digestive efficiency has also been noted in the sea urchin, albeit through a different mechanism, reduced stomach pH. These urchins exhibited a behavioural adjustment, to counteract this reduced efficiency. Fish may also adjust feeding behaviour in such instances, but broad behavioural impairments across various species with CO_2_ exposure^4^,^8^ may impact such processes and should be further investigated. An increased metabolic demand from ion transport processes, as seen in the present study, may detract from energy available for digestive functions in the intestine, slowing the process of digestion. Calculations using estimates of toadfish standard metabolic rate^43^ suggest that the energetic cost of CO_2_ acclimation in the intestine would account ~0.5% of whole animal O_2_ consumption^21^. Albeit small, any factor that promotes an energy reallocation or increases standard metabolic rate could exacerbate already narrow metabolic constraints^44^ or possibly interact with projected temperature elevations to increase overall impact^36^,^39^,^41^,^45^.

The demand to secrete HCO_3_^−^ in the intestine to maintain water balance is well-conserved across marine teleosts^30^. Increased intestinal HCO_3_^−^ secretion with CO_2_ exposure as reported here has also been demonstrated at higher CO_2_ levels in the plainfin midshipmen (~50,000 μatm CO_2_)^46^, in the toadfish (5000–20,000 μatm CO_2_)^23^, and suggested from gene expression and/or protein assays in the Japanese ricefish (7000 μatm CO_2_)^34^ and the Atlantic cod (1,200 and 2,200 μatm CO_2_)^41^. These studies suggest that increased intestinal HCO_3_^−^ secretion and metabolic demand during CO_2_ exposure could be a ubiquitous response to elevated CO_2_ throughout marine bony fishes. Finally, intestinal HCO_3_^−^ secretion results in formation and excretion of CaCO_3_ by marine fish which amounts to at least 3–15% of the marine inorganic CaCO_3_ production^24^. Although a study on the toadfish reported unaltered CaCO_3_ excretion rates^22^ at 1900 μatm a more recent study on toadfish^23^ and an earlier study on midshipmen^46^ may suggest otherwise. Thus, increased intestinal HCO_3_^−^ loss at elevated CO_2_ in other species may impact the magnitude of this globally important calcification process, a possibility worthy of further study. In this context, it is important to consider an alternative scenario in which the increase stimulation of HCO_3_^−^ secretion during CO_2_ exposure serves to assist the animal in buffering ingested acidified seawater, making conditions more favorable for carbonate precipitation and continued water absorption. However, this cannot be the sole purpose of the increase HCO_3_^−^ secretion, as the extra protons ingested in a given amount of 1900 μatm CO_2_ water is several orders of magnitude lower than the increase in HCO_3_^−^ secretion due to acclimation. These calculations support the idea that HCO_3_^−^ loss during CO_2_ exposure is not beneficial for the animal.

Surprisingly few studies have examined the impact of sustained global increases of carbon dioxide on acid-base and osmoregulatory processes in marine fish. While the presents study reports relatively small increases in bicarbonate secretion and oxygen consumption in the intestine, it is important to remember that these changes likely require reallocations or adjustments in an organism that may already be facing other downstream impacts of CO_2_. Although compensation for elevated CO_2_ in marine fish typically occurs within days^11^, it cannot be ruled out that longer acclimation periods, transgenerational effects, and/or adaptation may affect the dynamics of acid-base balance. Furthermore, interspecies differences associated with the cost of ionoregulatory demands are likely^40^.

## Methods

### Animal collection and care

Gulf toadfish (*Opsanus beta*) were obtained from local shrimpers as by-catch in Biscayne Bay and acclimated to flow-through, aerated, sand-filtered seawater from Bear Cut, FL (22–25 °C, 32–35 ppt) in the laboratory at the University of Miami for at least two weeks prior to experimentation. During this period, fish were fed squid twice weekly. Once introduced to experimental tanks, toadfish (HCO_3_^−^ secretion mass range: 21.7–30.9 g, O_2_ consumption mass range: 28.1–48.6 g) were fed 5% of their body weight weekly. Food was withheld at least 4 days prior to experimentation, a time period previously demonstrated to ensure that confounding effects of specific dynamic action (SDA) would not be a factor for HCO_3_^−^ secretion or oxygen consumption measurements^21^. Fish were sacrificed using a lethal dose of 0.2 g/L MS-222 buffered with 0.3 g/L NaHCO_3_. All general animal care and animal sacrifice protocols were carried out in accordance with relevant guidelines for experiments on teleost vertebrates provided by University of Miami IACUC (Institutional Animal Care and Use Committee). All experimental protocols were approved by University of Miami Institutional Animal Care and Use Committee. (IACUC 13-225). IACUC is accredited by the Association for Assessment and Accreditation of Laboratory Animal Care (AALAC). Toadfish were collected with the approval and in accordance with guidelines outlined by the Florida Fish and Wildlife Conservation Commission (Permit SAL-12-0729 SR).

### General experimental procedures

Toadfish were acclimated to either control or CO_2_ (~440 and ~1900 μatm CO_2_, respectively, Supplementary Table 2) for 2–4 weeks, a time period previously deemed sufficient to elicit a CO_2_ compensatory response in the toadfish^11^. Throughout the methods section, reference to CO_2_ refers to the 1900 μatm CO_2_ level, with respect to both treatment exposure and saline. Following acclimation, anterior intestinal tissue was dissected and immediately placed between two half-chambers where the tissue was bathed on either side by salines representative of *in vivo* conditions. Gut lumen-side (mucosal) salines were identical throughout all treatments, however, two different blood-side (serosal) salines (“control” or “CO_2_”) were used to mimic measured values at control or 1900 μatm CO_2_ (Supplementary Table 3)^11^. These Ussing chambers were combined with pH-stat titration to allow for measurement of HCO_3_^−^ secretion rates from the blood-side to the gut-side saline^29^. In addition, the O_2_ consumption rate of isolated intestinal segments were measured using a custom-built epithelial respirometer^21^. Each mounted tissue was treated with both the control and the CO_2_ serosal saline, allowing for examination of the effect of prior CO_2_ acclimation along with the effect of blood-side saline conditions that mimicked *in vivo* measured blood chemistry. If dynamic regulation was occurring in tissue from fish exposed to CO_2_ to reduce HCO_3_^−^ loss, reduced HCO_3_^−^ secretion rates would be expected in tissue from CO_2_ acclimated fish.

### Seawater manipulation

Stable CO_2_ levels were achieved using a Loligo pH-based negative feedback system (Loligo Systems, Tjele, Denmark) as previously described^11^,^22^. In this system, a standard curve was generated based on the relationship between known gas standards and seawater pH. Once a pH setpoint corresponding to 1900 μatm CO_2_ was calculated following calibration and measurement of ambient seawater, 100% CO_2_ was gassed directly into flow-through tanks via solenoid valves controlled by pH electrodes (Sentix H, wtw Germany) and a *p*CO_2_/pH DAQ-M digital relay instrument connected to CapCTRL software (Loligo Systems, Tjele, Denmark) to achieve the desired *p*CO_2_ setpoint. *p*CO_2_ levels typically stayed within 4–10 percent of setpoint values. Independent pH measurements were taken multiple times a week using a separate pH electrode (pH_NBS_, PHC3005, Radiometer, France). Target CO_2_ levels were confirmed with measurements of seawater TCO_2_ using a Corning 965 CO_2_ Analyzer (Corning 965, Corning Diagnostics, UK). TA (titratable alkalinity) and *p*CO_2_ levels were calculated from measured pH_NBS_ and TCO_2_ measurements into CO2SYS software^47^. These calculations confirmed target *p*CO_2_ values for control (ambient-~439 μatm CO_2_) and 1900 (1878 μatm CO_2_) were reached. Water chemistry parameters including temperature and salinity are reported in Supplementary Table 2.

### Electrophysiological measurements

In the Ussing chamber systems (Physiological Instruments, San Diego, CA, USA), current and voltage electrodes attached to an amplifier measured transepithelial potential (TEP, mV) differences between a baseline 0 μA current clamp and a 3 second pulse of 50 μA that was applied every 60s. Measurements were logged using Acknowledge software (v. 3.8.1, BIOPAC Systems). TEP values are reported with a luminal reference of 0 mV and conductance was calculated using Ohm’s law (Supplementary Table 1).

### *In vitro* Ussing chamber/pH stat electrophysiological and bicarbonate secretion measurements

Simultaneous measurement of electrophysiological parameters and bicarbonate secretion of isolated tissue was achieved using Ussing chamber systems (Physiological Instruments, San Diego, CA, USA) combined with automated pH-stat titration (TIM854/856 Titralab and Titramaster software v.5.1.0, Radiometer, Copenhagen, Denmark)^29^. Following acclimation in either control or CO_2_ treatment tanks (1900 μatm CO_2_, 2–4 weeks; Supplementary Table 2), anterior intestine segments were mounted on tissue holders designed to expose 0.71 cm^2^ of tissue to two half-chambers (1.6 mL) in the Ussing chamber system (P2400, Physiological Instruments). In this setup, isolated tissue was bathed in pre-gassed serosal (blood-side) or mucosal (gut-side) salines continuously mixed by air-lift gassing and held at 25 °C using a recirculating water bath. Mucosal saline composition remained unaltered throughout all experiments (Supplementary Table 3) and was gassed with 100% O_2_. “Control” and “CO_2_” serosal salines were designed to mimic *in vivo* HCO_3_^−^ and *p*CO_2_ levels previously measured in the plasma of toadfish during exposure to control and 1900 μatm CO_2_ (3.3 and 6.3 mM HCO_3_^−^, 0.225 and 0.462% CO_2_^11^, respectively; Supplementary Table 3).

A pH electrode (PHC4000-8, Radiometer, Denmark) and an acid-dispensing microburette were submerged into the mucosal half-chamber. The rate of acid titrant addition and the titrant concentration (0.005 N HCl) needed to hold the mucosal saline at a constant pH of 7.8 were used to calculate the epithelial HCO_3_^−^ secretion rate. Electrophysiological measurements were taken simultaneously with bicarbonate secretion rates (Supplementary Table 1). Once tissue preparations were considered stable based upon steady bicarbonate secretion and electrophysiological parameters, a minimum 30-minute measurement interval was recorded prior to a saline change. Each isolated intestinal tissue received both control and CO_2_ serosal saline treatments. Although tissues have been demonstrated to be viable for at least 5 hours in prior studies using this species and setup^29^, the order of serosal salines applied were randomized. During a saline change, the first serosal saline was carefully removed with a syringe, the half chamber was rinsed, and replaced by the second serosal saline treatment. Measurements post-saline change were continued until stable levels were recorded for a minimum of 30 minutes.

### *In vitro* oxygen consumption measurements

Following the same acclimation procedures outlined for bicarbonate secretion experiments, the oxygen consumption rate of isolated anterior intestine was measured using a custom-designed epithelial respirometer (Loligo Systems, Tjele, Denmark). Intestinal tissue was mounted so that 0.87 cm^2^ of tissue was exposed to two half-chambers (2.80 mL), mucosal saline on the gut side and one of two serosal saline treatments (described above) on the blood-side. All salines were pre-gassed with air (100% O_2_ saturation) rather than custom O_2_ mixes to make measurements of oxygen consumption rates comparable to whole-animal measurements. Saline HCO_3_^−^ concentrations were kept the same in the control and CO_2_ serosal salines (Supplementary Table 3).

Salines in half chambers were continuously mixed by micromagnetic glass-coated Teflon stir bars (Loligo Systems), and a Teflon tissue mount ensured that the system was gas-tight^21^. Oxygen measurements were conducted using a fiber-optic cable secured to the outside wall of either glass half-chamber that illuminated a fiber-optic sensor spot glued to the inside wall of each respective side. Each cable was connected to a separate single-channel oxygen meter (Fibox 3) used in conjunction with Oxy-View software (PST3-V6.02; PreSens, Regensburg, Germany). Prior to daily experiments, calibrations were performed with salines pre-gassed with air for 100% air saturation and gassed with N_2_ conditions representing no oxygen saturation.

Intermittent-flow respirometry was performed to determine oxygen consumption rates of isolated tissue by flushing and replacing salines using a manual gravity-fed system. Flush cycles guaranteed complete saline replacement during open cycles and time intervals during closed measurements were limited to ~20 minutes, to ensure that air saturation of tissue did not drop below 80%, since values below 75% were previously shown to restrict this tissue^21^. Previous use of this respirometry system has shown negligible rates of gas back-flux with the atmosphere or across chambers and a constant O_2_ consumption rate at air saturation above 75%^21^. Tissue O_2_ consumption was estimated from the mucosal and serosal O_2_ depletion rates.

### Statistical analysis

Two-way ANOVAs were used to analyze bicarbonate secretion (Fig. 1), oxygen consumption (Fig. 2, Supplementary Fig. 1), and electrophysiological measurements (Supplementary Table 1) with treatment exposure (acclimation) and saline exposure as independent variables. Significance for all tests was determined at P < 0.05 for all tests and all data are presented as means ± s.e.m. unless specifically noted otherwise.

## Additional Information

**How to cite this article**: Heuer, R. M. and Grosell, M. Elevated CO_2_ increases energetic cost and ion movement in the marine fish intestine. *Sci. Rep*. **6**, 34480; doi: 10.1038/srep34480 (2016).

## Supplementary Material

Supplementary Information
