# Supplementary material for: Elevated CO2 increases energetic cost and ion movement in the marine fish intestine
Source: Sci Rep. 2016 Sep 29;6:34480. doi: 10.1038/srep34480 (PMC5041088; doi:10.1038/srep34480)
Supplement: Supplementary Information [file srep34480-s1.pdf]

Supplementary information

"Elevated CO_2 increases energetic cost and ion movement in the marine fish intestine"

*Rachael M. Heuer and Martin Grosell

University of Miami- Rosenstiel School of Marine and Atmospheric Science,

4600 Rickenbacker Causeway, Miami, FL 33149

*Corresponding author: rheuer@rsmas.miami.edu, Phone 305-421-4665, Fax 305-421-4711

Current affiliation: University of North Texas, Department of Biological Sciences, 1511 West Sycamore, Denton, TX 76203

Supplementary Figure 1: Mass normalized Oxygen consumption of isolated anterior tissue from control and CO_2 acclimated toadfish

Effect of blood-side saline composition and acclimation exposure on oxygen consumption rates (means ± s.e.m.) of isolated anterior intestinal tissue taken from toadfish acclimated to control (~440 μatm CO_2; n=12) or ~1900 μatm CO_2 (n=8) for 2-4 weeks. Tissues from either control or 1900 μatm CO_2 acclimated fish mounted in this dual-chambered epithelial respirometer were bathed on either side by salines designed to mimic *in vivo* ionic composition. Each tissue received two blood-side serosal saline treatments, control saline and CO_2 saline, that were representative of HCO_3 and previously measured in toadfish blood following acclimation at control and 1900 μatm CO_2 (Supplementary Table 2). Two-way ANOVA, Acclimation exposure: $P<0.005$, Saline $P<0.743$, Acclimation exposure x saline: $P<0.829$, *significant acclimation effect.

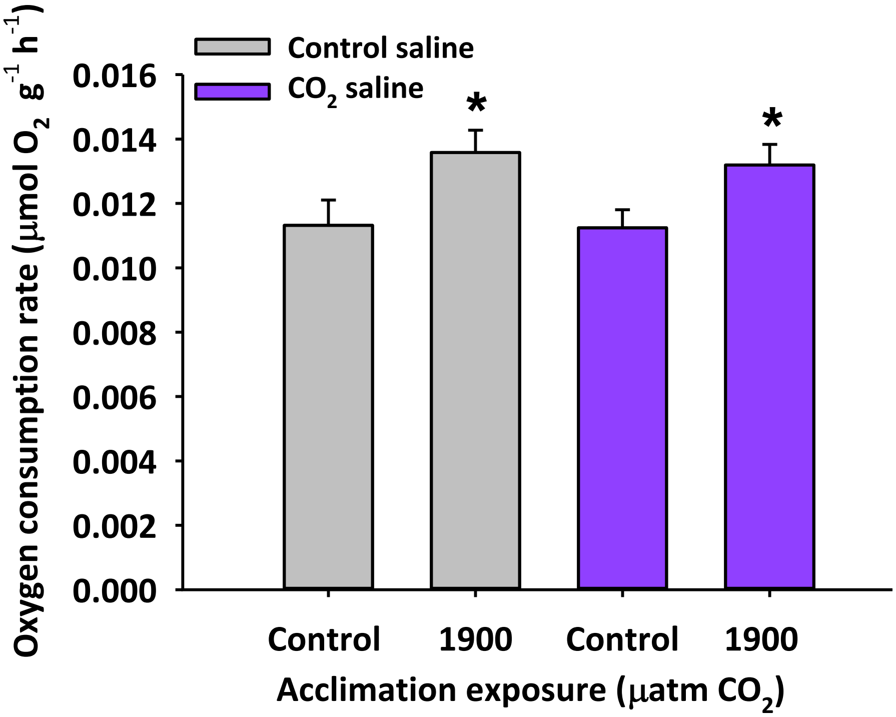

Supplementary Table 1: Electrophysiological measurements from isolated toadfish intestinal tissue

Fish acclimation and serosal saline type	Transepithelial potential (TEP) (mV)	Conductance (μSi)
Anterior control tissue		
Control serosal saline	-17.71 ± 1.38	9.78 ± 0.24
CO_2 serosal saline	-18.06 ± 0.71	9.97 ± 0.21
Anterior CO_2 tissue		
Control serosal saline	-17.63 ± 0.98	10.34 ± 0.27
CO_2 serosal saline	-16.73 ± 1.35	10.16 ± 0.31

Values are means ± s.e.m. See Supplementary Table 3 for control and CO_2 saline compositions

Supplementary Table 2: Water chemistry parameters. Values are presented as means ± standard deviation.

	pH_{NBS}	pCO_2 (μatm)	Alkalinity (μmol kg^{-1})	TCO_2 (μmol kg^{-1})	Salinity (PSU)	Temperature (°C)
Control (ambient)	8.15 ± .03	439 ± 24	2294 ± 42	2037 ± 31	34.1 ± 0.5	22.5 ± 1.1
1900 μatm CO_2	7.58 ± .02	1878 ± 54	2334 ± 41	2303 ± 40	34.2 ± 0.5	22.5 ± 1.2

Supplementary Table 3: Serosal saline composition used in isolated tissue experiments for both Ussing/pH stat and oxygen consumption measurements

Constituent	Serosal		Mucosal
	Control serosal	CO_2 serosal	
NaCl (mmol $^{-1}$)	151	151	69
KCl (mmol $^{-1}$)	3	3	5
$MgSO_4$ (mmol $^{-1}$)	0.88	0.88	77.5
$MgCl_2$ (mmol $^{-1}$)	-	-	22.5
Na_2HPO_4 (mmol $^{-1}$)	0.5	0.5	-
KH_2PO_4 (mmol $^{-1}$)	0.5	0.5	-
$CaCl_2$	1	1	5
Hepes, free acid	3	3	-
Hepes, sodium salt	3	3	-
Urea	4.5	4.5	-
Glucose	5	5	-
Osmolality (mOsm l $^{-1}$)	320**	320**	320**
pH	7.8†	7.8†	7.8
Gas††	0.225% CO_2 in O_2*	0.462% CO_2 in O_2*	O_2
HCO_3^- (mmol $^{-1}$)	3.30*	6.33*	-

*Values designed to mimic measured or calculated pCO_2 and HCO_3^- values in gulf toadfish exposed to control or ~1900 μatm CO_2[1]
**Adjusted with mannitol to ensure transepithelial isomostic conditions in all experiments.
†pH 7.8 was maintained by pH-stat titration
††Salines gassed for at least one hour prior to experimentation. All salines in O_2 consumption setup were gassed with air.

1 Esbaugh, A. J., Heuer, R. & Grosell, M. Impacts of ocean acidification on respiratory gas exchange and acid-base balance in a marine teleost, *Opsanus beta*. *J.Comp Physiol B* **182**, 921-934, doi:10.1007/s00360-012-0668-5 (2012).
